# Atomoxetine reduces hyperactive/impulsive behaviours in neurokinin-1 receptor ‘knockout’ mice

**DOI:** 10.1016/j.pbb.2014.10.008

**Published:** 2014-12

**Authors:** Katharine Pillidge, Ashley J. Porter, Temis Vasili, David J. Heal, S. Clare Stanford

**Affiliations:** a Department of Neuroscience, Physiology and Pharmacology, University College London, Gower St, London WC1E 6BT, UK; b RenaSci Ltd, BioCity, Pennyfoot Street, Nottingham, NG1 1GF, UK

**Keywords:** ADHD, Attention Deficit Hyperactivity Disorder, 5-CSRTT, 5-Choice Serial Reaction-Time Task, LDEB, Light/dark exploration box, NK1R, Neurokinin-1 receptor, VITI, Variable inter-trial interval, Atomoxetine, Attention Deficit Hyperactivity Disorder, 5-Choice Serial Reaction-Time Task, Hyperactivity, Impulsivity, NK1R^−/−^ ‘knockout’ mouse

## Abstract

**Background:**

Mice with functional ablation of the neurokinin-1 receptor gene (NK1R^−/−^) display behavioural abnormalities which resemble the hyperactivity, inattention and impulsivity seen in Attention Deficit Hyperactivity Disorder (ADHD). Here, we investigated whether the established ADHD treatment, atomoxetine, alleviates these abnormalities when tested in the light/dark exploration box (LDEB) and 5-Choice Serial Reaction-Time Task (5-CSRTT).

**Methods:**

Separate cohorts of mice were tested in the 5-CSRTT and LDEB after treatment with no injection, vehicle or atomoxetine (5-CSRTT: 0.3, 3 or 10 mg/kg; LDEB: 1, 3 or 10 mg/kg).

**Results:**

Atomoxetine reduced the hyperactivity displayed by NK1R^−/−^ mice in the LDEB at a dose (3 mg/kg) which did not affect the locomotor activity of wildtypes. Atomoxetine (10 mg/kg) also reduced impulsivity in NK1R^−/−^ mice, but not wildtypes, in the 5-CSRTT. No dose of drug affected attention in either genotype.

**Conclusions:**

This evidence that atomoxetine reduces hyperactive/impulsive behaviours in NK1R^−/−^ mice consolidates the validity of using NK1R^−/−^ mice in research of the aetiology and treatment of ADHD.

## Highlights

• We compared the behavioural response to atomoxetine in NK1R^−/−^ and wildtype mice.
• Atomoxetine reduced hyperactivity and impulsivity in NK1R^−/−^ mice but not wildtypes.
• This was not explained by changes in animals' emotional status or motor motivation.
• NK1R^−/−^ mice are more sensitive to atomoxetine (an ADHD treatment) than wildtypes.
• These findings consolidate the NK1R^−/−^ mouse model of ADHD.

## 1 Introduction

Atomoxetine (Strattera®: atomoxetine hydrochloride) is a preferential noradrenaline reuptake inhibitor, which inhibits the noradrenaline (NAT), serotonin (SERT) and dopamine (DAT) transporters with *K*_i_ values of 5, 77 and 1451 nM, respectively (Bymaster et al., 2002). Although the drug was first developed as an antidepressant, it is now an approved treatment for Attention Deficit Hyperactivity Disorder (ADHD) (Preti, 2002) and is marketed as a non-psychostimulant alternative to the first-line ADHD treatments, amphetamine and methylphenidate.

ADHD is characterised by three core abnormalities: excessive impulsivity, inattention and hyperactivity. Mice lacking functional neurokinin-1 receptors (NK1R^−/−^) typically display ADHD-like inattention and impulsivity when tested in the 5-Choice Serial Reaction-Time Task (5-CSRTT) (Yan et al., 2011). They are also hyperactive in a number of procedures (Fisher et al., 2007, Herpfer et al., 2005, Yan et al., 2010). Moreover, low doses of NK1R antagonists induce hyperactivity in wildtype mice (Yan et al., 2010) and exacerbate inattention and impulsivity in the 5-CSRTT (Weir et al., 2014).

The ADHD treatments, amphetamine, methylphenidate and guanfacine, all reduce the hyperactivity of these mice (Pillidge et al., 2014, Yan et al., 2010). Although amphetamine did not reduce the impulsivity/inattention displayed by NK1R^−/−^ mice in the 5-CSRTT, the non-stimulant guanfacine, did (Pillidge et al., 2014, Yan et al., 2011). The proposal that these mice could be used to study the aetiology of ADHD-like behaviours in vivo is supported by evidence that *TACR1* (the human equivalent of the NK1R gene) polymorphisms are associated with ADHD (Sharp et al., 2009, Sharp et al., 2014, Yan et al., 2010).

The effects of atomoxetine in the 5-CSRTT in outbred rodents are remarkably consistent. In Long Evans and Lister-hooded rats, this drug reduces premature responses (impulsivity) (Fernando et al., 2012, Paterson et al., 2011, Robinson, 2012, Robinson et al., 2008) but has negligible effects on omissions (inattention). The same pattern has even been reported in zebrafish performing a modified version of the 5-CSRTT: atomoxetine attenuated premature responses, whereas omissions were unaffected (Parker et al., 2014). However, whenever atomoxetine does increase omissions, this is generally paralleled by increased response latencies (Baarendse and Vanderschuren, 2012, Sun et al., 2012), suggesting a drug effect on arousal or motivation to carry out the task.

Our aim in these experiments was to explore further the use of NK1R^−/−^ mice as a preclinical resource for investigating ADHD-like behaviour. To that end, we investigated whether atomoxetine ameliorates hyperactivity of NK1R^−/−^ mice and/or deficits in their cognitive performance, in the light–dark exploration box (LDEB) and 5-CSRTT, respectively.

## 2 Methods

### 2.1 Ethics statement

All experiments were authorised under the Animals (Scientific Procedures) Act, 1986 (UK) and were approved by the Ethical Review Panel at University College London. This report was written in concordance with the ARRIVE guidelines for animal experiments (Kilkenny et al., 2010).

### 2.2 Drugs

Tomoxetine (atomoxetine) hydrochloride was purchased from Sigma Aldrich, UK, dissolved in 0.9% saline and injected intraperitoneally (i.p.) in a volume of 10 mL/kg. Doses of 1, 3 and 10 mg/kg were tested in the LDEB, with each mouse tested with one dose, only. In the 5-CSRTT, doses of 0.3, 3 and 10 mg/kg were tested at once-weekly intervals in the same animals, with every animal receiving each dose once.

### 2.3 Animals

NK1R^−/−^ mice and their wildtype counterparts were bred at University College London in a facility held at 21 ± 2 °C, 45 ± 5% humidity, with a 12:12 h light: dark cycle (07.00–19.00 h). The home-cages incorporated environmental enrichment (cardboard tunnels and nesting material (LBS Biotechnology, UK)) and were cleaned twice-weekly (bedding obtained from Litaspen Premium (Lillico)). Rodent chow was obtained from Harlan UK (2018 global Rodent Diet). All the mice derived from inbred homozygous strains (see: Yan et al., 2010, Pillidge et al., 2014) and were of a 129/Sv × C57BL/6J background, backcrossed with an outbred MF1 strain many generations ago (de Felipe et al., 1998).

### 2.4 Light/dark exploration box

NK1R^−/−^ and wildtype mice, from inbred homozygous lines, were used to enable comparison of the results of this study with those from our previously published reports (Dudley et al., 2013, Pillidge et al., 2014, Yan et al., 2010). Both genotypes were tested in the light/dark exploration box (LDEB) after either no injection (NI), or administration of vehicle (0.9% saline, 10 mL/kg) or atomoxetine (1, 3 or 10 mg/kg, i.p.) (N = 5 per group). The choice of drug doses was informed by published reports of its effects on the behaviour of rodents (e.g. (rat) Robinson et al., 2008; (mouse) Balci et al., 2008). The LDEB also served as a dose-range study to determine the most appropriate doses to use in the 5-CSRTT. Treatments were allocated in a counterbalanced sequence, with each mouse receiving only one treatment. One wildtype and one NK1R^−/−^ mouse were always tested simultaneously, with the same treatment, in adjacent LDEBs. The procedure is described fully in Fisher et al. (2007) and Herpfer et al. (2005). In brief, the mice were habituated to the test room for at least 3 h and then confined, individually, to the dark zone (4 lx) of the LDEB for 60 min, after which they were injected with their allocated treatment, or left untreated and, replaced in the dark zone for a further 30 min. After a total of 90 min in the dark zone, the mice were transferred, individually, to the light zone (20 lx) and allowed to commute freely between the two zones. Their behaviour was recorded by a digital video camera and scored later by a blinded observer. The first 10 min of activity after transfer to the light zone was used in the statistical analysis. One NK1R^−/−^ mouse from the vehicle group was excluded from the analysis, because it was an outlier in nearly every behavioural measure.

### 2.5 5-Choice Serial Reaction-Time Task (5-CSRTT)

The 5-CSRTT protocol followed that detailed previously (Yan et al., 2011). This 5-CSRTT study was part of a larger experiment investigating whether the behavioural phenotype of adult mice was influenced by breeding strategy. This involved a comparison of the behaviour of homozygous wildtype (NK1R^+/+^) and NK1R^−/−^ progeny of inbred homozygous parents (‘*homs*’) with the same genotypes bred from heterozygous (NK1R^+/−^) breeding pairs (‘*hets*’). Wildtype and NK1R^−/−^ *homs* were housed separately, but *het* mice were housed in cages that contained at least one wildtype and one NK1R^−/−^ mouse. Each home-cage contained 2–4 mice. 12 wildtype male and 12 NK1R^−/−^ male mice were used at 6–8 weeks of age (weighing WT *hom*: 26.5–33.5 g, NK1R^−/−^ *hom*: 28.3–32.8 g, WT *het*: 29.9–36.0 g, NK1R^−/−^ *het*: 29.5–35.7 g) at the start of training.

The mice were brought into the laboratory on weekdays between 09.00 and 09.30 h and weighed before training/testing in the 5-CSRTT between 10.00 h and 12.00 h (AM session) or 13.00 h and 15.00 h (PM session). Mice were fed, after the 5-CSRTT sessions had ended, with a quota of food adjusted to maintain subjects at 90% of their original free-feeding body weight. Water was available ad libitum.

The apparatus (Med Associates, St. Albans, VT, USA) comprised sound-attenuated operant chambers with five equally-spaced apertures incorporated into one wall. Apertures could be illuminated independently and mouse ‘nose-pokes’ into the apertures interrupted infrared beams, which enabled scoring of correct, incorrect, premature and perseverative responses. Omitted responses were scored when no nose-poke occurred. Correct responses were rewarded with sweetened milk (0.01 mL of 30% condensed milk solution), delivered into a magazine in the opposite wall. Collection of the reward (nose-pokes into the magazine) initiated the next trial. Incorrect, omitted and premature responses were punished with a 5 s time-out, during which the house-light was extinguished and no new trial could be initiated. Perseverative responses were not punished.

Mice were assigned to one of four test chambers in a fully counterbalanced design and were tested in the same chamber throughout. They were first habituated to the apparatus before graduating through increasingly challenging stages of training (see: Yan et al., 2011 for the criteria for each stage). After reaching a stable baseline at Stage 6, for 3 consecutive days, mice qualified for drug testing. They were tested with a variable inter-trial interval (VITI; 2, 5, 10 or 15 s, delivered on a random schedule) once weekly, on Fridays, only if they had maintained a stable baseline of responding in the intervening Tuesday–Thursday training sessions. VITI tests were used, rather than unexpected prolongation of the ITI, because the former prevents the strategic use of interval-timing, thereby increasing the required cognitive effort. The first week of testing was carried out with treatment-naïve mice to compare the baseline performance of the two genotypes from the two breeding colonies (no -injection 1: ‘NI-1’, to be reported in full elsewhere).

Over the following 5 weeks, mice were tested 30 min after an injection of either vehicle (saline, 10 mL/kg), or atomoxetine (0.3, 3 or 10 mg/kg), or after another ‘no-injection’ session (‘NI-2’). This NI-2 session served as a control for any effects of repeated testing on baseline performance (see: Weir et al., 2014). The doses of atomoxetine used in this experiment were based on the findings from the LDEB: specifically, a lower dose (0.3 mg/kg) replaced the 1 mg/kg dose, used in the LDEB, in order to test whether any cognitive effects of the drug could be dissociated from a reduction in locomotor activity. Each mouse received each treatment once, only. The sessions were counterbalanced across subjects, using a pseudo William's Latin-square, to account for any effects of repeated testing and potential nuisance factors (e.g. time of day: see Weir et al. (2014) and Yan et al. (2011)). Test sessions lasted for 45 min, or 100 trials, whichever occurred first. Two wildtypes (one *hom* and one *het*) and one NK1R^−/−^ (*hom*) mouse failed to graduate through training in the 5-CSRTT, and were not tested.

### 2.6 Statistics

Statistical analyses were performed, using InVivoStat (Clark et al., 2012), on raw or transformed data (arcsine, log10 or square-root): whichever optimised the homogeneity of variance in the ‘predicted versus residuals’ plot in InVivoStat. The ‘normal probability plot’ was used to determine whether or not the data were normally distributed. If not, a rank transformation was applied: i.e. the data were assigned ranks, as for a non-parametric analysis, which were subjected to parametric tests.

In the LDEB, two-way ANOVAs were performed on raw or transformed data, with the main factors ‘Genotype’ and ‘Treatment’. First, the ANOVA compared the factors across all groups (uninjected, vehicle and drug-treated). If there was a main effect of either factor, or an interaction between them, further analyses were carried out using *post hoc* ANOVA to compare vehicle controls with drug treatment (main effect of ‘Drug’). LSD tests were used as further *post hoc* comparisons of individual groups.

Repeated-measures analyses were used to examine data from the 5-CSRTT. These used a mixed model approach: ‘*Genotype*’ *and* ‘*Colony*’ were first used as between-subjects factors and ‘*Drug*’ was the within-subjects factor. There was no interaction between *Colony* and *Drug* for any variable and so the groups for the ‘*Colony*’ factor were collapsed. ‘*Time of day*’ (i.e. AM session/PM session) was also investigated because previous studies indicated that this can influence behaviour in the 5-CSRTT (Weir et al., 2014, Yan et al., 2011). When *Time of day* had a main effect, or interacted with genotype, it was used as a blocking factor, to account for any additional variance in the data. This was valid since there were no *Time of day* ∗ *Drug* interactions for any dependent variable (i.e. drug treatment had the same effect on behaviour regardless of *Time of day*). When there were no effects of *Time of day*, this factor was collapsed across all subjects. A main effect of ‘*Genotype*’ or ‘*Drug*’, or an interaction between them, was used as the criterion for carrying out *post hoc* pairwise comparisons.

## 3 Results

### 3.1 Atomoxetine reduces motor activity in wildtype and NK1R^−/−^ mice (Fig. 1)

**Fig. 1:**
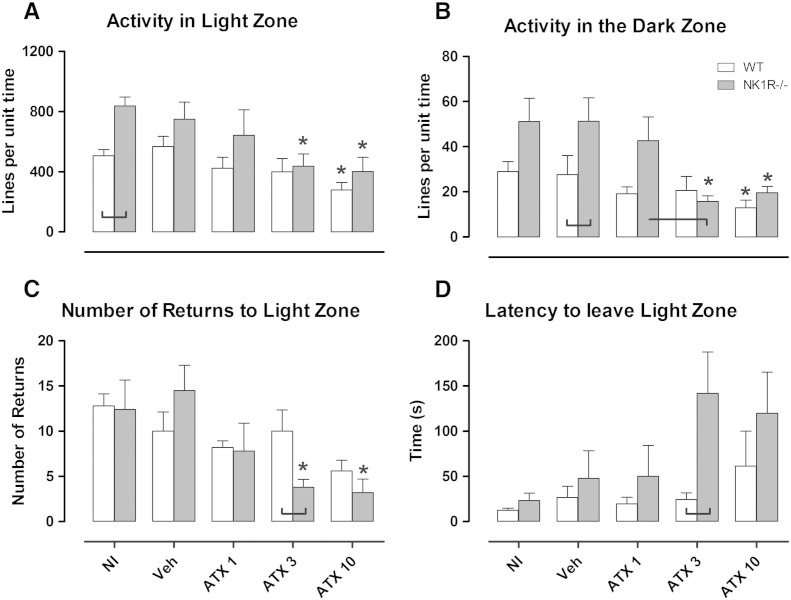
The effects of atomoxetine (1, 3 and 10 mg/kg, i.p.), vehicle (saline) or no injection (NI) on A: activity per unit time in the light zone, B: activity per unit time in the dark zone, C: number of returns to the light zone and D: latency to leave the light zone in wildtype (white bars) and NK1R^−/−^ mice (grey bars) in the light–dark exploration box. Data show mean ± SEM. *P < 0.05 versus vehicle within genotype. Lines linking bars indicate statistical significance of P < 0.05. N = 5.

Uninjected NK1R^−/−^ mice were hyperactive in the light zone [[RAW]geno: F_(1,15)_ = 13.69, P = 0.002, WT vs. KO, NI: P = 0.004, Fig. 1A] and vehicle-treated NK1R^−/−^ mice were hyperactive in the dark zone [[RAW]geno: F_(1,15)_ = 7.06, P = 0.018, WT vs. KO, VEH: P = 0.044, Fig. 1B].

Atomoxetine reduced the activity of mice of both genotypes in the light zone [[RAW]drug: F_(3,31)_ = 3.84, P = 0.019] and dark zone [[LOG10]drug: F_(3,29)_ = 4.69, P = 0.009]. Specifically, the mid-range dose of atomoxetine (3 mg/kg) reduced the activity of NK1R^−/−^ mice, but not wildtypes, in the light [KO VEH vs. ATX3: P = 0.039] and dark zones [KO VEH vs. ATX 3: P = 0.003]. However, the higher dose (10 mg/kg) reduced locomotor activity of both genotypes in both zones [light: VEH vs. ATX10, WT: P = 0.043, KO: P = 0.023; dark: VEH vs. ATX10, WT: P = 0.039, KO: P = 0.021], compared to vehicle.

Atomoxetine also caused an overall reduction in the *number of returns* to the light zone [[LOG10]drug: F_(3,31)_ = 4.13, P = 0.014, Fig. 1C], across both genotypes [[LOG10]drug ∗ geno: F_(3,31)_ = 1.66, P = 0.196]. However, pairwise comparisons revealed that this reduction was evident only in NK1R^−/−^ mice at 3 mg/kg [KO VEH vs. ATX3: P = 0.008] and 10 mg/kg [KO VEH vs. ATX10: P = 0.001]. The *number of returns* to the light zone (a measure of passive avoidance) by wildtypes was not reduced by any dose of drug. Across all doses, atomoxetine had no effect on the *latency to leave* the light zone (active avoidance) [[LOG10]drug: F_(3,29)_ = 1.78, P = 0.174, Fig. 1D], but just missed the statistical criterion for increasing the latency of NK1R^−/−^ mice at 3 mg/kg [KO VEH vs. ATX3: P = 0.078]. However, a genotype difference [[LOG10]geno: F_(3,29)_ = 6.68, P = 0.015] was evident at 3 mg/kg [ATX3, WT vs. KO: P = 0.021]. Atomoxetine had no effect on *time spent in the light zone* [[RAW]drug: F_(3,31)_ = 0.09, P = 0.964 (data not shown)].

### 3.2 Atomoxetine attenuates premature responding by NK1R^−/−^ mice but not wildtypes

**Fig. 2:**
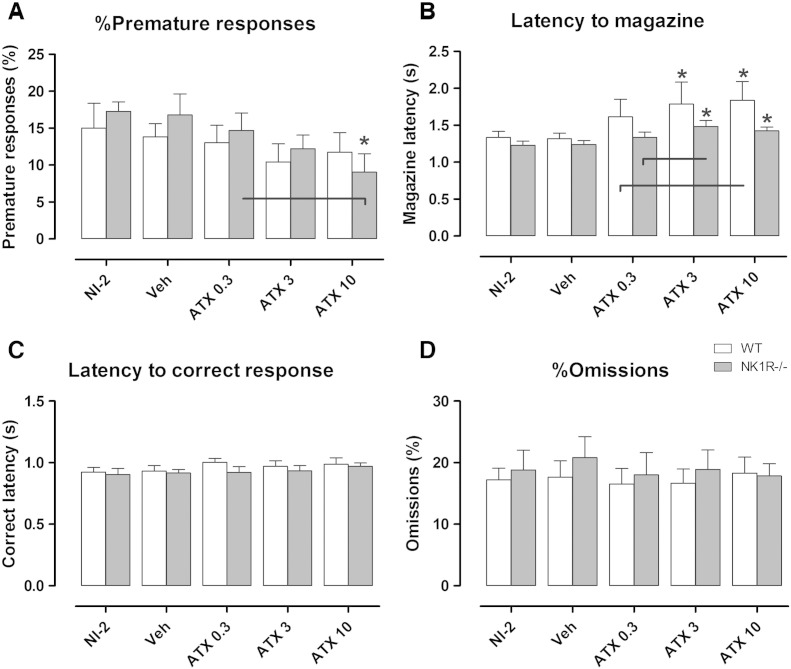
A: The effects of atomoxetine (0.3, 3 and 10 mg/kg, i.p.) on A: %premature responses, B: latency to magazine, C: latency to correct response and D: %omissions compared with vehicle (saline) and no injection (NI-2), in wildtype (white bars) and NK1R^−/−^ mice (grey bars). Data show mean ± SEM. N = 9–11. Lines linking bars indicate statistical significance of P < 0.05, * indicates P < 0.05 versus vehicle within genotype.

In this experiment, NK1R^−/−^ mice did not display an impulsive phenotype: i.e. there was no difference between the genotypes in the rate of premature responding overall [[SQRT]geno: F_(1,13)_ = 0.44, P = 0.520, Fig. 2A] or at baseline (NI-2) [WT vs. KO: P = 0.336]. Across all doses, atomoxetine caused a reduction in %*premature responses* [[SQRT]drug: F_(3,55)_ = 2.89, P = 0.044], which did not depend on genotype [[SQRT]drug ∗ geno: F_(3,55)_ = 0.76, P = 0.521]. Nevertheless, *post hoc* analysis revealed that the drug reduced *premature responses* by NK1R^−/−^ mice, only, at 10 mg/kg [KO, VEH vs. ATX10: P = 0.006].

### 3.3 Atomoxetine prolongs the latency to reward, but not latency to correct response

Wildtype and NK1R^−/−^ mice took the same length of time to respond correctly [[RANK]geno: F_(1,19)_ = 0.60, P = 0.448, Fig. 2B] and to reach the magazine, overall [[RANK]geno: F_(1,19)_ = 0.74, P = 0.400, Fig. 2C]. Atomoxetine increased the *latency to magazine* [[RANK]drug: F_(3,55)_ = 10.59, P < 0.001]: this affected both genotypes at the two higher doses [VEH vs. ATX3, WT: P = 0.033, KO: P < 0.001; VEH vs. ATX10, WT: P < 0.001, KO: P < 0.001]. However, the drug had no effect on the *latency to correct response* [[RAW]drug: F_(4,74)_ = 1.85, P = 0.128]. Another measure of motivation, the *number of trials* completed (Table 1), was unaffected by either genotype [[ARCSINE]geno: F_(1,13)_ = 0.89, P = 0.363] or drug [[ARCSINE]drug: F_(4,51)_ = 0.89, P = 0.479].

**Table 1:** The effects of atomoxetine on behaviour of NK1R^−/−^ (‘KO’) and wildtype (‘WT’) mice in the 5-CSRTT. Values show mean ± SEM. N = 9–11 per group.

	No injection		Vehicle		Atomoxetine 0.3 mg/kg		Atomoxetine 3 mg/kg		Atomoxetine 10 mg/kg	
Behaviour	WT	KO	WT	KO	WT	KO	WT	KO	WT	KO
*Total number of trials*	100.0 ± 0	100.0 ± 0	100.0 ± 0	100.0 ± 0	100.0 ± 0	100.0 ± 0	100.0 ± 0	100.0 ± 0	99.6 ± 0.4	99.2 ± 0.6
%*Accuracy*	97.5 ± 0.85	97.2 ± 0.86	96.9 ± 1.00	97.3 ± 0.56	97.3 ± 1.02	97.4 ± 0.57	98.0 ± 0.79	97.8 ± 0.51	97.1 ± 1.07	97.2 ± 0.77
*Perseveration* (per 100 trials)	12.3 ± 3.51	12.1 ± 4.05	12.2 ± 3.78	17.3 ± 7.00	19.1 ± 11.57	13.1 ± 4.15	12.3 ± 4.86	10.5 ± 3.86	17.2 ± 6.49	15.6 ± 5.23

### 3.4 Atomoxetine has no effect on accuracy, omission errors or *perseveration*

Time of day affected %*accuracy*, %*omissions* and *perseveration*, but there was no interaction with drug treatment, so ‘*time of day*’ was used as a blocking factor in the analysis of these behaviours. There was no overall genotype difference in %*omissions* [[SQRT] geno: F_(1,18)_ = 0.03, P = 0.865, Fig. 2D], %*accuracy* [[RAW]geno: F_(1,18)_ = 0.06, P = 0.816, Table 1] or *perseveration* [LOG10]geno: F_(1,18)_ = 0.00, P = 0.979, Table 1]. Moreover, atomoxetine had no effect on any of these behaviours [%*accuracy*: [RAW] F_(4,74)_ = 0.24, P = 0.914; %*omissions* [SQRT] F_(4,74)_ = 0.27, P = 0.897; *perseveration* [LOG10] F_(4,74)_ = 0.99, P = 0.418].

## 4 Discussion

Here we report that atomoxetine reduced ADHD-like hyperactive/impulsive behaviours in NK1R^−/−^ mice, at doses that did not affect wildtypes. Although interactions between genotype and drug were not statistically significant, *post*-*hoc* comparisons revealed that NK1R^−/−^ mice were more sensitive to this drug and/or responded more consistently than the wildtypes. The findings broadly replicate those reported on tests of this drug in outbred rats performing the 5-CSRTT. However, to the best of our knowledge, this is the first study, using the 5-CSRTT, to find that atomoxetine is more effective in rodents expressing behavioural abnormalities, analogous to those seen in ADHD patients, than in wildtypes.

### 4.1 Atomoxetine prevents hyperactivity

Uninjected NK1R^−/−^ mice were hyperactive in the light zone of the LDEB, but this was not evident after they had experienced an i.p. injection. Interestingly, the reverse was observed in the dark zone. The reason for this is not clear, but influences of the functional status of NK1R on the response to stress have been well documented (for reviews see: Ebner and Singewald, 2006, Stanford, 2014).

Atomoxetine reduced the locomotor hyperactivity displayed by NK1R^−/−^ mice in the LDEB at a dose (3 mg/kg) that did not affect wildtypes. Despite a reduction in the *number of returns to the light zone* (increased passive avoidance), this reduction in locomotor activity response is unlikely to be explained by an increase in anxiety-like behaviour (see: Stanford, 2007) because there was a concomitant increase in *latency to leave the light zone* (reduced active avoidance) at this dose. Moreover, there was no change in the *time in the light zone*, which would be expected if there was an appreciable effect on animals' emotionality.

The genotype difference in the locomotor response to atomoxetine is consistent with its effects in other rodent models of ADHD, including spontaneously hypertensive (SHR) rats, trimethyltin chloride-treated (TMT) rats (Tamburella et al., 2012) and 6-OHDA-lesioned rats (Moran-Gates et al., 2005), but is at odds with a report that the drug did not affect hyperactive DAT-KO mice (Del'Guidice et al., 2014). Also, it is striking that NK1R^−/−^ mice were more sensitive to the α2-adrenoceptor agonist, guanfacine, in this test (Pillidge et al., 2014).

### 4.2 Atomoxetine reduces impulsivity

Unlike our previous studies (Dudley et al., 2013, Pillidge et al., 2014, Yan et al., 2011), NK1R^−/−^ mice did not display an excessively impulsive phenotype at baseline (NI-2) in this study. This is likely to be because *premature responses* of NK1R^−/−^ mice dissipate on repeated testing (Weir et al., 2014). Also, this behaviour seems to be influenced by an interaction between NK1Rs and environmental/epigenetic factors arising from the breeding strategy (to be reported elsewhere). Nevertheless, atomoxetine (10 mg/kg) reduced *premature responses* by NK1R^−/−^ mice, regardless of breeding strategy, but had no effect in wildtypes. However, an increase in the *latency to magazine* was evident in both genotypes, suggesting that different mechanisms regulate these two behaviours. Importantly, neither of these behavioural changes is likely to be explained by any drug induced reduction in appetite, or other motivation to carry out the task, because there was no corresponding increase in %*omissions* or *latency to correct response*, which are well-established measures of motivation.

These results are supported by consistent reports that atomoxetine reduces impulsivity in outbred Lister-hooded rats performing the 5-CSRTT (Baarendse and Vanderschuren, 2012, Robinson et al., 2008), especially in animals that display high impulsivity at baseline (i.e. High Impulsive (HI) rats) (Blondeau and Dellu-Hagedorn, 2007, Fernando et al., 2012, Tomlinson et al., 2014). Moreover, atomoxetine is more effective when impulsivity is increased by manipulating the task parameters: for example, by extending the inter-trial interval (ITI) (Baarendse and Vanderschuren, 2012, Paterson et al., 2011). However, Robinson (2012) also reports that atomoxetine is efficacious under baseline conditions and when the task is made more difficult (e.g. by reducing the stimulus duration or adding a distracter stimulus). To date, only one study has examined the effect of atomoxetine in the SHR (the most extensively characterised rodent model of ADHD) in the 5-CSRTT (Dommett, 2014). Atomoxetine (0.1–5 mg/kg) did not affect any behavioural measure at any dose, but the protocol used in that study differed substantially from that used here.

Although we have not studied the mechanisms underlying these behavioural responses to atomoxetine, our earlier study similarly revealed a genotype difference in sensitivity to guanfacine in the same behavioural tests (Pillidge et al., 2014). This suggests a genotype difference in regulation of noradrenergic transmission, possibly mediated by α2-adrenoceptors: a proposal supported by neurochemical evidence (Fisher et al., 2007, Herpfer et al., 2005, see also: Stanford, 2014). If so, it is not surprising that the effects of atomoxetine, which has a high and selective affinity for the noradrenaline transporter, and for which changes in extracellular transmitter depend on neuronal firing-rate (i.e. are impulse dependent: Bymaster et al., 2002), differ in NK1R^−/−^ and wildtype mice. This possibility that the noradrenergic response to atomoxetine differs in NK1R^−/−^ and wildtype mice merits further research. However, in light of evidence that NK1R influence the permeability of the blood brain barrier (Harford-Wright et al., 2014), a pharmacokinetic explanation is also possible.

Although atomoxetine preferentially inhibits the noradrenaline transporter, the selectivity of this drug is likely to be reduced at high doses. Microdialysis studies have reported that the concentration of extracellular noradrenaline and dopamine is increased in the prefrontal cortex of mice following administration of either 1 or 3 mg/kg of this drug (Koda et al., 2010) but no such increase was reported for serotonin. This disparity in monoamine responses suggests that, although atomoxetine has a *K*_i_ for SERT that is only 10-fold higher than that for the noradrenaline transporter, serotonin is unlikely to be directly involved in the behavioural effects of this drug.

### 4.3 Atomoxetine does not improve attention

Atomoxetine did not improve either measure of attention (%*omissions* and %*accuracy*) in either genotype. There are isolated reports that atomoxetine improves accuracy in rats (Baarendse and Vanderschuren, 2012, Robinson, 2012) but, in general, %*omissions* in the 5-CSRTT increase, rather than diminish, after atomoxetine treatment (e.g. Baarendse and Vanderschuren, 2012, Sun et al., 2012). However, inattentiveness is consistently reduced by atomoxetine in clinical studies (Faraone and Glatt, 2010, Hazell et al., 2011, Wilens et al., 2006), suggesting that this aspect of behaviour in the 5-CSRTT might not translate reliably between rodents and humans.

## 5 Conclusions

The findings reported here are consistent with preclinical and clinical evidence that atomoxetine causes a robust reduction in locomotor hyperactivity and impulsivity, especially in animals that display a high incidence of these behaviours. We believe that this is the first study to report that atomoxetine is more effective in mice that express behavioural abnormalities in the 5-CSRTT that resemble those in ADHD, when compared with their wildtypes. Our findings further lead us to infer that atomoxetine would be most suitable for treating the hyperactive–impulsive subtype of ADHD, particularly in a subset of patients with *TACR1* polymorphisms.

## Author contributions

Conceived study: SCS, DJH. 5-CSRTT (training): KP, AJP, TV (unregulated procedures, only). 5-CSRTT (testing): KP. LDEB: KP. Analysis of data: KP, SCS. Preparation of manuscript: KP, SCS (with editorial input from other authors).
